# Metabolomics reveals the mechanism of action of meropenem and amikacin combined in the treatment of *Pseudomonas aeruginosa*

**DOI:** 10.3389/fcimb.2023.1327452

**Published:** 2023-12-05

**Authors:** Hai Yang, Zhen Huang, Jiali Yue, Jianqi Chen, Mingming Yu, Chengming Qu

**Affiliations:** ^1^ Affiliated Qingdao Central Hospital of Qingdao University, Qingdao Cancer Hospital, Qingdao, China; ^2^ School of Medicine and Pharmacy, Ocean University of China, Qingdao, China

**Keywords:** *Pseudomonas aeruginosa*, metabolomics, meropenem, amikacin, LC-MS/MS

## Abstract

The treatment of *Pseudomonas aeruginosa* infection often involves the combined use of β-lactam and aminoglycoside antibiotics. In this study, we employed metabolomic analysis to investigate the mechanism responsible for the synergistic activities of meropenem/amikacin combination therapy against multidrug-resistant *P. aeruginosa* strains harboring OXA-50 and PAO genes. Antibiotic concentrations for meropenem (2 mg/L) monotherapy, amikacin (16 mg/L) monotherapy, and meropenem/amikacin (2/16 mg/L) combination therapy were selected based on clinical breakpoint considerations. Metabolomic analysis revealed significant alterations in relevant metabolites involved in bacterial cell membrane and cell wall synthesis within 15 min of combined drug administration. These alterations encompassed various metabolic pathways, including fatty acid metabolism, peptidoglycan synthesis, and lipopolysaccharide metabolism. Furthermore, at 1 h and 4 h, the combination therapy exhibited significant interference with amino acid metabolism, nucleotide metabolism, and central carbon metabolism pathways, including the tricarboxylic acid cycle and pentose phosphate pathway. In contrast, the substances affected by single drug administration at 1 h and 4 h demonstrated a noticeable reduction. Meropenem/amikacin combination resulted in notable perturbations of metabolic pathways essential for survival of *P. aeruginosa*, whereas monotherapies had comparatively diminished impacts.

## Introduction

Antibiotic resistance is an increasingly serious threat to global health. *Pseudomonas aeruginosa* has attracted serious public concerns due to its adaptability, diversity, and high resistance rate (Behzadi et al., 2022; Algammal et al., 2023). *P. aeruginosa* exhibits remarkable adaptability in diverse environments and is commonly associated with conditions such as ventilator-associated pneumonia, cystic fibrosis, diabetes, and severe liver and kidney failure, and is a leading cause of morbidity and mortality (Pang et al., 2019; Ahmed, 2022). In recent years, the emergence of multi-drug resistant (MDR) *P. aeruginosa* has posed a grave threat to public health (Mirzaei et al., 2020). MDR in *P. aeruginosa* is defined as the resistance to at least one antibiotic from each of the three major classes: aminoglycosides, quinolones, and cephalosporin (Barbier and Wolff, 2010; Kunz Coyne et al., 2022).

Aminoglycosides are often used to treat infections due to *P. aeruginosa*, but aminoglycoside-resistant *P. aeruginosa* has been known as early as the 1960s (Poole, 2005; Farhan et al., 2021). Carbapenem antibiotics (β-lactam class) have emerged as highly effective antimicrobial agents against Gram-negative bacteria (Meletis et al., 2012). The resistance mechanisms to meropenem and aminoglycosides include the combination of potential reductions in outer membrane permeability, up-regulated expression of efflux pump genes, and the production of metallo-β-lactamases (MBL) (Hassuna et al., 2020). In clinical practice, the use of aminoglycosides as monotherapy has been associated with increased mortality rates (Leibovici et al., 1997; Avent et al., 2022). Consequently, combination therapy involving the concomitant administration of aminoglycosides and β-lactam antibiotics is commonly employed (Nakamura et al., 2000; Craig, 2011; Mahmoud et al., 2021). *In vitro* synergistic effects of β-lactam and aminoglycoside combination in the treatment of *P. aeruginosa* have been demonstrated (Song et al., 2003; Jung et al., 2004). This article aims to understand the potential mechanism of action of amikacin and meropenem combination responsible for their synergistic activities from the perspective of metabolomics.

## Materials and methods

### Wet lab section

#### Antibiotics, reagents and bacterial isolates

**Figure 1:**
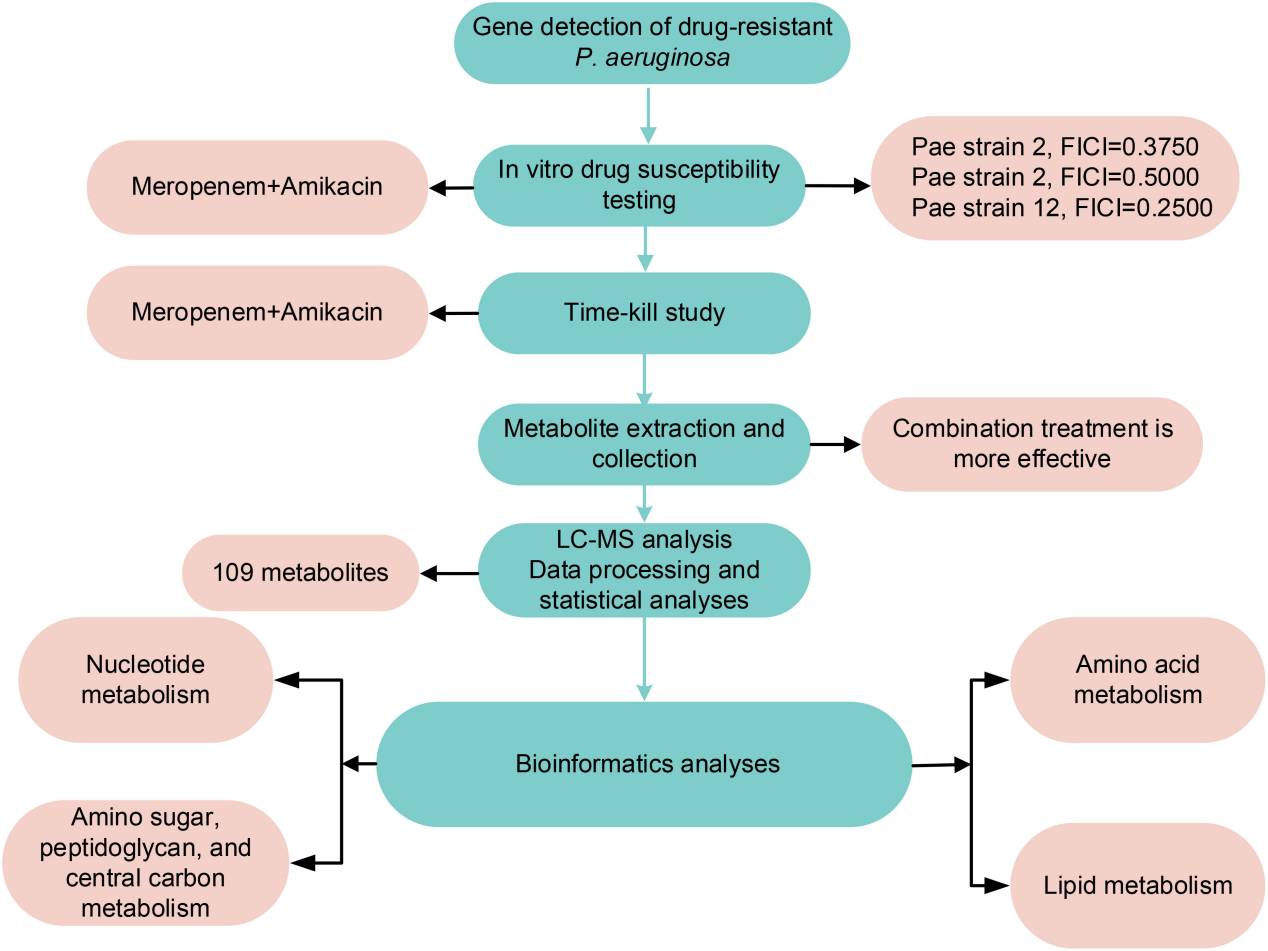
Materials and Methods and Results flow chart.

Meropenem and amikacin (Shanghai McLean Biochemical Co. Ltd. Shanghai, China) solutions were prepared. The two antibiotics were dissolved in pure water to achieve a concentration of 5210 μg/mL and stored at -80°C. Prior to use, the working solution was further diluted with Milli-Q water (Australian Northern Rye Millipole) and subjected to filtration. Three isolates of *P. aeruginosa* obtained from the Affiliated Hospital of Qingdao University were cultivated in cation-adjusted Mueller-Hinton broth (CAMHB; Land Bridge, Beijing, China). *E. coli* ATCC 25922 and *P. aeruginosa* ATCC 27853 were selected as quality control strains. The β-lactam resistance genes carried by clinical isolate were determined by next-generation sequencing as previously described (Feng et al., 2021; Zhu et al., 2022a). The method flow chart is shown in **Figure 1**.

#### *In vitro* susceptibility testing

Broth microdilution method based on Clinical and Laboratory Standards Institute (CLSI) guidelines (Clsi, 2020) was utilized to determine the minimum inhibitory concentration (MIC) values of meropenem and amikacin, both individually and in combination. Stock solutions of meropenem and amikacin were diluted in a gradient fashion using Mueller-Hinton broth (MHB) medium. The resulting solutions were then dispensed into sterile 96-well plates. The concentrations for meropenem and amikacin ranged from 0.25 mg/L to 128 mg/L for each antibiotic. The turbidity of *P. aeruginosa* was assessed; the bacterial suspension was adjusted to the density of a 0.5 McFarland standard and subsequently diluted to a final concentration of 5 × 10^5^ cfu/mL in the sterile 96-well plates. The 96-well plates were incubated at 37°C for 20 h before being examined for their respective MIC. The MIC was defined as the concentration of antibiotics wherein no visible bacterial growth was observed.

The antibacterial effect of the combination antibiotics was evaluated using the checkerboard method, and the fractional inhibitory concentration index (FICI) was calculated using the following equation and criteria.


$$\begin{array}{c} FICI=\frac{MIC\;of\;antibiotic\;A\;in\;combination}{MIC\;of\;antibiotic\;A\;alone}\\ +\frac{MIC\;of\;antibiotic\;B\;in\;combination}{MIC\;of\;antibiotic\;B\;alone} \end{array}$$

Synergy: FICI ≤ 0.5; additive effect: 0.5<FICI<1; irrelevant effect: 1<FICI<2; antagonism: FICI>2.

#### Time-kill study

*P. aeruginosa* strain 12 was used for the time-kill study and inoculated into a 15 mL Eppendorf tube containing 10 mL of MHB medium. The tube was then placed in a constant temperature incubator, rotating at a speed of 180 rpm/min, with a temperature set at 37°C, and allowed to cultivate overnight. The bacterial density was grown to logarithmic phase of approximately 10^8^ CFU/mL, which is equivalent to a normalized OD_600_ value of 0.5. The concentrations of meropenem and amikacin were selected according to their clinical breakpoints, and the bacterial cultures were divided into four groups: the control group without any antibiotics, meropenem (2 mg/L) alone, amikacin (16 mg/L) alone, and the combination of meropenem and amikacin (2 mg/L and 16 mg/L). Each treatment group contained five replicates, which were cultured in a constant-temperature incubator with shaking.

#### Metabolites extraction method

*P. aeruginosa* samples were taken for metabolite extraction at 15 min, 1 h, and 4 h respectively, and centrifuged at 3220×*g*, 4°C for 10 min (Zhu et al., 2022b). After the supernatant was discarded, the remaining residue was washed with 1 mL of pre-cooled normal saline medium twice. 500 μL solution containing 1 μM internal standard 3-[(3-cholamidopropyl)-dimethylamino]-1-propanesulfonic acid (CHAPS), N-cyclohexyl-3-aminopropanesulfonic acid (CAPS), piperazine-N,N′-bis(2-ethanesulfonic acid) (PIPES), and Tris in chloroform-methanol-water (1:3:1) cold solutions was added. The mixture was quickly frozen in liquid nitrogen, thawed naturally on ice, and the freezing and thawing process was repeated 3 times to release metabolites from the cells. The sample was centrifuged at 3220×*g* and 4°C for 10 minutes to remove cell debris. Three hundred microliters of supernatant was transferred to a 1.5 mL Eppendorf tube, centrifuge at 14000×*g* and 4°C for 10 minutes. 200 μL of the supernatant was put in a vial for metabolite analysis. Obtain quality control samples by taking 10 µL of each of the above samples and ensuring thorough and even mixing.

#### LC-MS analysis

The liquid chromatography-mass spectrometry (LC-MS) detection methods were optimized based on prior studies (Zhu et al., 2022b; Zhang et al., 2023; Zhu et al., 2023). Sample analysis was conducted using the Ultimate 3000 ultra-high performance liquid chromatography (UHPLC) system (Thermo Scientific, San Jose, CA, USA) coupled with the Q-Exactive Orbitrap mass spectrometer (Thermo Scientific, San Jose, CA, USA) utilizing a resolution of 35,000. The detection range spanned from *m/z* 50 to 1250 Da, and the ion source employed both positive and negative electrospray ionization (ESI) modes. For chromatographic separation, a HILIC column (2.1 × 100 mm, 1.7 μm; ACE 1.7 μm, HILIC-A, UK) was utilized, with the column temperature set at 40°C. The mobile phase consisted of a 10 mM ammonium carbonate aqueous solution (mobile phase A) and acetonitrile (mobile phase B). The flow rate was set at 0.3 mL/min, while the injection volume was 10 μL. The gradient elution program initiated with 80% mobile phase B and transitioned to 20% B over the first 15 min. This was followed by a 3-minute elution with 5% B and a final equilibration step with 80% B for 8 minutes.

### Dry lab section

#### Data processing, bioinformatics, and statistical analyses

The raw data were processed and analyzed by the software Progenesis QI (Waters, USA). Metabolites were identified by retention time and *m/z* from the LC-MS results, and metabolite intensities were normalized by log_10_-transformed values with automatic scaling. Statistical analysis was performed using the MetaboAnalyst 5.0 metabolomics analysis website (https://www.metaboanalyst.ca/). Principal component analysis (PCA) was performed on each treatment group at 15 min, 1 h, and 4 h. Student’s t-test (P<0.05), fold difference (FC) ≥ 2 (log_2_FC ≥ 1 or ≤ -1) was used to identify metabolites with significant differences. Metabolite identification and metabolic pathway analysis were performed using Kyoto Encyclopedia of Genes and Genomes (KEGG) and Human Metabolome Database (HMDB) databases (Behzadi and Ranjbar, 2019).

## Results

### *In vitro* susceptibility testing

The three *P. aeruginosa* clinical isolates carried drug resistance genes, OXA-50 and PAO (**Table 1**). The MIC range of amikacin alone against the above strains is 2-16 mg/L; the MIC range of meropenem alone is 4-16 mg/L. According to CLSI (Clsi, 2020), the interpretive criteria for amikacin against *P. aeruginosa* are as follow: ≤16 mg/L (susceptible), 32 mg/L (intermediate), and ≥64 mg/L (resistant); for meropenem are the following: ≤2 mg/L (susceptible), 4 mg/L (intermediate), and ≥8 mg/L (resistant). These strains exhibited antibiotic resistance to meropenem. When meropenem and amikacin were used in combination, the MIC values of meropenem and amikacin against the three strains dropped to below the respective breakpoints, and the FICI were less than 0.5, showing a synergistic effect. According to the results of the drug susceptibility test, isolate 12 with the lowest FICI was selected to further study metabolomic changes in response to drug treatment.

**Table 1:** Minimum inhibitory concentration (MIC) of amikacin alone, meropenem alone and amikacin/meropenem combination against *P. aeruginosa*, as well as drug resistance genes encoded in each isolate.

Strains	drug resistance genes encoded	MIC (mg/L)			FICI
		Amikacin	Meropenem	Amikacin/Meropenem	
Control					
*E.coli* ATCC 25922		2	1	–	–
*P. aeruginosa* ATCC 27853		4	1	–	–
Pae strain 2^a^	aph(3’)-IIb, fosA, catB7, blaOXA-50,blaPAO	2	16	0.5/2	0.3750
Pae strain 8^b^	crpP, aph(3’)-IIb, fosA, catB7, blaOXA-50, blaPAO	8	4	2/1	0.5000
Pae strain 12^c^	aph(3’)-IIb, fosA, sul2, catB7, blaOXA-50, blaPAO	16	8	2/1	0.2500

MIC, minimum inhibitory concentration; FICI, fractional inhibitory concentration index; CLSI breakpoints for interpretation of amikacin MIC results: ≤16mg/l (susceptible), 32mg/L (intermediate), and≥64mg/L (resistant); and meropenem MIC results: ≤2mg/L (susceptible), 4mg/L (intermediate), and≥8mg/L (resistant) for P. aeruginosa.

^a^ *P. aeruginosa* strain 2.

^b^ *P. aeruginosa* strain 8.

^c^ *P. aeruginosa* strain 12.

### Changes in metabolomics induced by amikacin and meropenem alone or in combination

**Figure 2:**
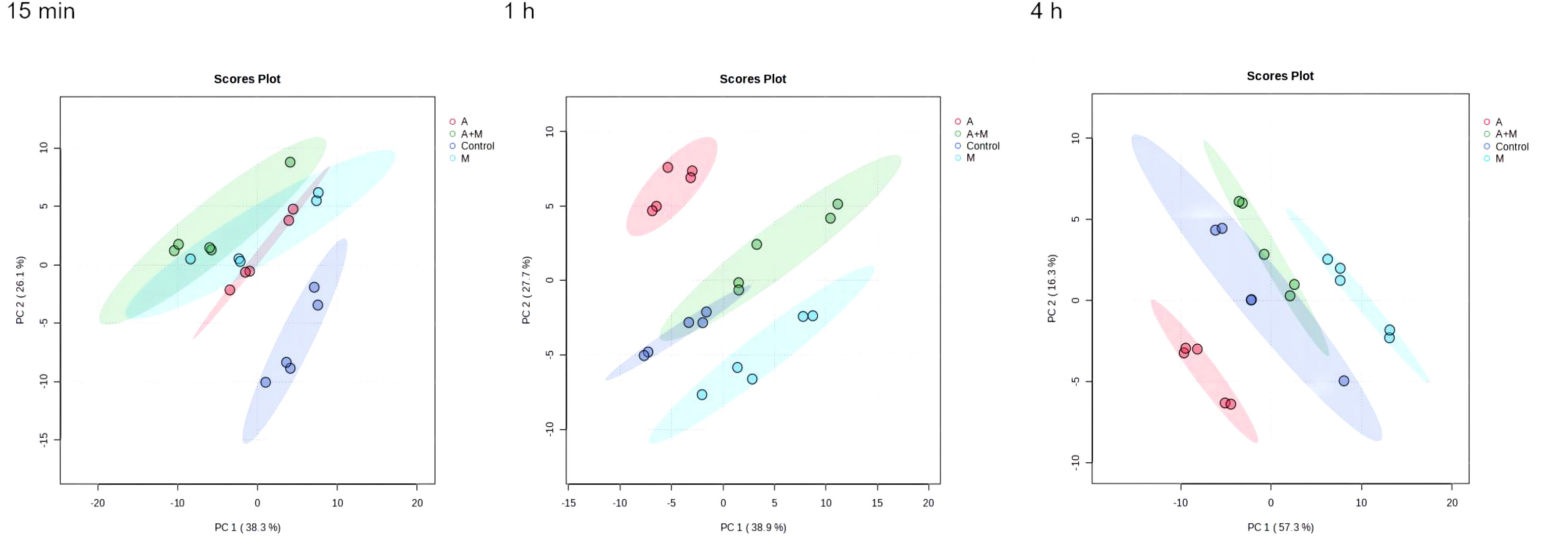
Principal component analysis (PCA) plots of metabolite levels of *P. aeruginosa* in the control group (Control), amikacin group (A), meropenem group (M) and amikacin/meropenem combination group (A+M) at 15 min, 1 h and 4 h.

The flow chart of the results for this study is shown in **Figure 1**. Metabolic analysis of *P. aeruginosa* 12 isolate using LC-MS has revealed changes in 109 metabolites associated with key metabolic pathways. Principal component analysis (PCA) was utilized to delineate the distinct effects of drug treatments on the metabolic changes of *P. aeruginosa*. The findings demonstrated that the combined treatment group exhibited significant dissimilarity compared to both the single treatment group and the control group across various time points (**Figure 2**). A notable disparity emerged between monotherpies and combination therapy, as well as the control group, after 15 min of antibiotic exposure. Subsequently, at 1 h and 4 h post-administration, the distinction between the single-drug treatment group and the control group diminished, displaying partial overlap between the meropenem and the control group at 1 h, while the group treated with combination therapy remained substantially distant from the control group.

**Figure 3:**
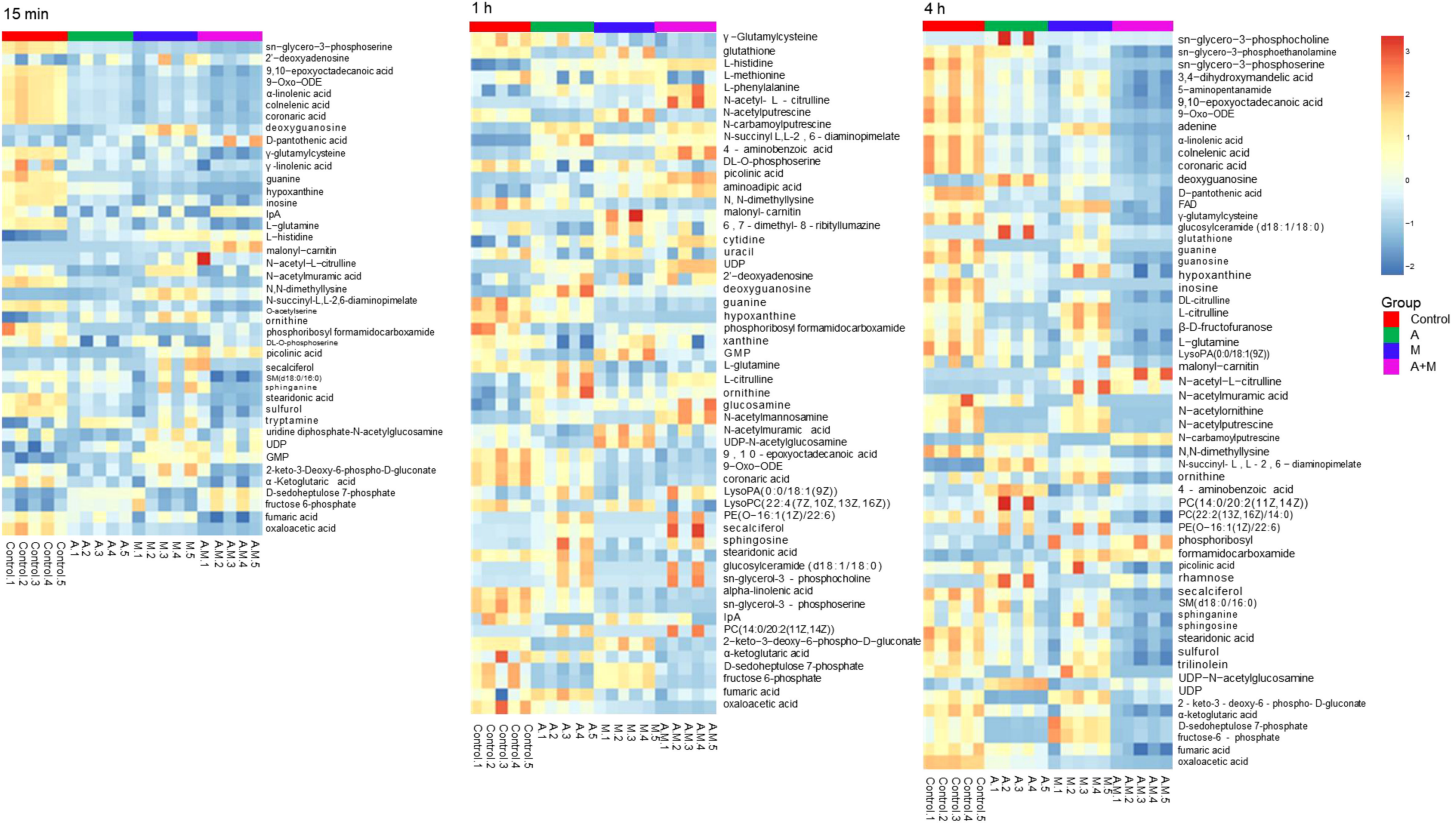
Heatmap of amikacin group (A), meropenem group (M) alone and amikacin/meropenem combination (A+M) against *P. aeruginosa* strains at 15 min, 1 h and 4 h.

Heatmap visualization of the results demonstrated temporal changes in the impacted metabolites of *P. aeruginosa* for different antibiotic groups (**Figure 3**). Amikacin and meropenem alone or in combination interfered with various metabolic pathways such as amino acids, nucleotides, central carbon metabolism, lipids, and peptidoglycan, as summarized in **Table 2**.

**Table 2:** Sequence of metabolomic changes in *P. aeruginosa* following amikacin and meropenem as monotherapy and combination therapy.

Time	Amikacin/Meropenem	Amikacin	Meropenem
15 min	**Cell wall synthesis** ↓ (1.52) N-acetylmuramic acid; ↓(1.26) UDP-GlcNAc	**Cell wall synthesis** ↓ (2.52) N-acetylmuramic acid; ↓ (6.57) UDP-GlcNAc	**Cell wall synthesis** ↑ (1.55) N-acetylmannosamine
	**Outer membrane** ↓ (10.4) sn-glycero-3-phosphoserine; ↓ (5.30) α-linolenic acid	**Outer membrane** ↓ (2.91) α-linolenic acid; ↓ (3.11) sn-glycero-3-phosphoserine	**Outer membrane** ↓ (3.38) α-linolenic acid; ↓ (4.96) sn-glycero-3-phosphoserine
	**Amino acid metabolic pathway** ↓ (2.97) γ-glutamylcysteine; ↑ (3.05) L-histidine; ↑ (150) N-acetyl-L-citrulline; ↑ (5.69) N-succinyl-L,L-2,6-diaminopimelate; ↓ (2.42) O-acetylserine;↑ (3.18) picolinic acid; ↓ (5.03) N,N-dimethyllysine; ↑ (396) malonyl-carnitin; ↑ (2.02) ornithine	**Amino acid metabolic pathway** ↑ (2.11) L-histidine; ↑ (7.39) malonyl-carnitin; ↑ (29.3) N-acetyl-L-citrulline; ↓ (2.19) DL-O-phosphoserine; ↓ (3.03) N,N-dimeth yllysine; ↑ (2.16) tryptamine; ↑ (2.13) ornithine	**Amino acid metabolic pathway** ↑ (2.97) L-histidine; ↑ (84.3) N-acetyl-L-citrulline; ↑ (10.9) N-succinyl-L,L-2,6-diaminopimelate; ↑ (2.95) ornithine; ↑ (2.21) picolinic acid; ↓ (3.13) N,N-dimethyllysine; ↑ (42.0) malonyl-carnitin
	**Nucleotide, nicotinate and nicotinamide pathway** ↑ (2.39) GMP; ↓ (7.20) guanine; ↓ (139) hypoxanthine; ↑ (2.12) UDP; ↓ (3.09) FAICAR; ↓ (3.34) L-glutamine; ↓ (2.52) inosine; ↓(2.06) sulfurol; ↑ (2.18) D- pantothenic acid	**Nucleotide, nicotinate and nicotinamide pathway** ↓ (4.26) guanine; ↓ (8.56) FAICAR	**Nucleotide, nicotinate and nicotinamide pathway** ↑ (2.08) UDP; ↑ (2.53) 2’-deoxyadenosine; ↑ (2.59) deoxyguanosine; ↑ (2.49) GMP; ↓ (4.41) guanine; ↓ (5.90) hypoxanthine; ↓ (15.1) FAICAR
	**Central carbon metabolism** ↓ (1.72) α-ketoglutaric acid; ↓ (5.95) oxaloacetic acid; ↑ (5.48) D-sedoheptulose 7-P; ↑ (3.05) fructose 6-P	**Central carbon metabolism** ↓ (2.47) oxaloacetic acid; ↑ (4.24) D-sedoheptulose 7-P; ↑ (2.42) fructose 6-P	**Central carbon metabolism** ↓ (3.22) oxaloacetic acid; ↑ (1.84) D-sedoheptulose 7-phosphate
	**Lipid metabolism pathway** ↓ (3.30) colnelenic acid; ↑ (3.28) secalciferol; ↓ (2.49) SM(d18:0/16:0); ↓ (2.08) sphinganine; ↓ (3.28) PE(16:0/16:0)	**Lipid metabolism pathway** ↓ (2.31) colnelenic acid; ↑ (1.99)secalciferol	**Lipid metabolism pathway** ↓ (2.52) colnelenic acid; ↑ (5.95) secalcifero(l
1 h	**Cell wall synthesis** ↓ (1.22) N-acetylmuramic acid; ↓ (3.20) UDP-GlcNAc	**Cell wall synthesis** ↑ (0.41) N-acetylmuramic acid; ↑ (0.25) UDP-GlcNAc	**Cell wall synthesis** ↑ (1.53) N-acetylmuramic acid; ↑ (2.31) UDP-GlcNAc
	**Outer membrane** ↑ (71.9) sn-glycero-3-phosphocholine; ↓ (2.49) α-linolenic acid; ↓ (5.65) sn-glycero-3-phosphoserine	**Outer membrane** ↑ (67.3) sn-glycero-3-phosphocholine; ↑ (1.79) phosphatidylethanolamine	**Outer membrane** ↓ (2.35) α-linolenic acid; ↓ (3.95) sn-glycero-3-phosphoserine; ↓ (9.76) sn-glycero-3-phosphocholine
	**Amino acid metabolic pathway** ↓ (2.01) γ-glutamylcysteine; ↓ (13.0) glutathione; ↑ (2.64) L-histidine; ↓ (2.47) L-methionine; ↑ (2.10) L-phenylalanine; ↑ (293) N-acetyl-L-citrulline; ↓ (3.21) N-acetylputrescine; ↑ (8.40)N-carbamoylputrescine; ↑ (10.8) N-succinyl-L,L-2,6-diaminopimelate; ↑ (6.93) 4-aminobenzoic acid; ↓ (2.30) DL-O-phosphoserine; ↑ (5.50) picolinic acid; ↑ (2.29) aminoadipic acid; ↓ (2.52) N,N-dimethyllysine; ↑ (23.7) malonyl-carnitin	**Amino acid metabolic pathway** ↑ (2.02) L-histidine; ↑ (0.08) glutathione; ↑ (38.9) N-acetyl-L-citrulline; ↑ (0.21) N-acetylputrescine; ↑ (8.80) N-carbamoylputrescine; ↑ (13.3) N-succinyl-L,L-2,6-diaminopimelate; ↑ (3.63) 4-aminobenzoic acid; ↑ (0.35) DL-O-phosphoserine ↑ (2.23) L-citrulline; ↑ (2.52) ornithine	**Amino acid metabolic pathway** ↑(2.05) L-histidine; ↑ (30.1) N-acetyl-L-citrulline; ↑ (10.1) N-succinyl-L,L-2,6-diaminopimelate; ↑ (2.56) picolinic acid; ↓ (2.27) N,N-dimethyllysine; ↑ (73.4) malonyl-carnitin
	**Nucleotide, nicotinate and nicotinamide pathway** ↑ (2.27) cytidine; ↑ (10.0) UDP; ↑ (2.40) 2’-deoxyadenosine ↑ (2.31) deoxyguanosine; ↓ (2.96) guanine; ↓ (806) hypoxanthine; ↓ (2.53) uracil	**Nucleotide, nicotinate and nicotinamide pathway** ↑ (5.85) UDP; ↑ (0.43) xanthine; ↑ (0.25) GMP; ↑ (4.78) deoxyguanosine; ↑ (0.29) uracil; ↑ (2.17) FAICAR	**Nucleotide, nicotinate and nicotinamide pathway** ↑ (2.61) deoxyguanosine; ↓ (2.61) guanine; ↓ (11.6) hypoxanthine; ↓ (2.06) L-glutamine; ↓ (2.17) FAICAR
	**Central carbon metabolism** ↑ (2.23) glucosamine; ↓ (5.33) oxaloacetic acid; ↓ (3.99) D-sedoheptulose 7-P; ↓ (2.32) D-gluconate-6-P	**Central carbon metabolism** ↑ (1.51) glucosamine; ↑ (0.12)D-sedoheptulose 7-P; ↑ (0.38) D-gluconate-6-P	**Central carbon metabolism** ↑ (1.70) glucosamine; ↓ (3.04) oxaloacetic acid;
	**Lipid metabolism pathway** ↑ (3.42) LysoPA(0:0/18:1(9Z)); ↓ (2.81) LysoPC(22:4); ↑ (18.5) PE(O-16:1(1Z)/22:6); ↑ (2.14) sphingosine; ↑PE(16:0/16:0); ↑ (24.6) glucosylceramide (d18:1/18:0); ↑ (29.6) PC(14:0/20:2(11Z,14Z))	**Lipid metabolism pathway** ↑ (3.18) LysoPA(0:0/18:1(9Z)); ↑ (0.47) LysoPC(22:4); ↑ (22.4) PE(O-16:1(1Z)/22:6); ↑ (2.51) sphingosine; ↑ (PE(16:0/16:0); ↑ (20.9) glucosylceramide (d18:1/18:0); ↑ (29.6) PC(14:0/20:2(11Z,14Z))	**Lipid metabolism pathway** ↓ (5.75) PE(O-16:1(1Z)/22:6); ↓ (8.07) glucosylceramide (d18:1/18:0); ↓ (2.15) PC(14:0/20:2(11Z,14Z))
4 h	**Cell wall synthesis** ↑ N-acetylmuramic acid; ↓UDP-GlcNAc	**Cell wall synthesis** ↓ (1.99) N-acetylmuramic acid; ↓ (7.21) UDP-GlcNAc	**Cell wall synthesis** ↓ (3.55) N-acetylmuramic acid
	**Outer membrane** ↑ (4.01) sn-glycero-3-phosphocholine; ↓ (3.13) phosphatidylethanolamine; ↓ (4.68) α-linolenic acid	**Outer membrane** ↓ (2.11) α-linolenic acid; ↑ (121) sn-glycero-3-phosphocholine	**Outer membrane** ↓ (2.29) α-linolenic acid; ↑ (5.59) sn-glycero-3-phosphocholine
	**Amino acid metabolic pathway** ↓ (4.03) γ-glutamylcysteine; ↓ (2.24) 3,4-dihydroxymandelic acid; ↓ (2.64) 5-aminopentanamide; ↑ (118) N-acetyl-L-citrulline; ↓ (6.84) N-acetylputrescine; ↑ (6.38) N-carbamoylputrescine; ↑ (6.91) N-succinyl-L,L-2,6-diaminopimelate ↑ (2.89) picolinic acid; ↓ (4.03) N,N-dimethyllysine; ↑ (97.7) malonyl-carnitin; ↓ (6.84) N-acetylornithine; ↓ (22.2) L-citrulline; ↓ (3.11) ornithine	**Amino acid metabolic pathway** ↓ (127) glutathione; ↑ (23.6) N-acetyl-L-citrulline; ↓ (6.37) N-acetylputrescine; ↑ (6.54) N-carbamoylputrescine; ↑ (15.4) N-succinyl-L,L-2,6-diaminopimelate; ↑ (2.21) 4-aminobenzoic acid; ↑ (2.58) malonyl-carnitin; ↓ (9.72) N-acetylornithine; ↓ (5.36) L-citrulline	**Amino acid metabolic pathway** ↓ (2.07) γ-glutamylcysteine; ↑ (169) N-acetyl-L-citrulline; ↑ (10.0) N-succinyl-L,L-2,6-diaminopimelate; ↑ (2.81) picolinic acid; ↑ (23.6) malonyl-carnitin
	**Nucleotide, nicotinate and** **nicotinamide pathway** ↓ (2.81) adenine; ↓ (4.54) guanine; ↓ (18257)hypoxanthine; ↑ (2.60) FAICAR; ↓ (4.20) L-glutamine; ↓ (2.55) guanosine; ↓ (3.03) inosine; ↓ (3.02) FAD; ↓ (2.24) sulfurol	**Nucleotide, nicotinate and** **nicotinamide pathway** ↑ (4.53) UDP; ↑ (3.93) deoxyguanosine; ↓ (3.68) hypoxanthine	**Nucleotide, nicotinate and** **nicotinamide pathway** ↑ (2.15) deoxyguanosine; ↓ (2.18) guanine; ↓ (36.2) hypoxanthine; ↓ (2.01) L-glutamine
	**Central carbon metabolism** ↓ (2.10) α-ketoglutaric acid; ↓ (2.16) fumaric acid; ↓ (6.54) oxaloacetic acid; ↓ (7.80) D-sedoheptulose 7-P; ↓ (10.8) fructose 6-P	**Central carbon metabolism** ↓ (2.16) oxaloacetic acid; ↓ (15.5) D-sedoheptulose 7-P; ↓ (32.2) fructose 6-P	**Central carbon metabolism** NA
	**Lipid metabolism pathway** ↓ (3.23) colnelenic acid; ↓ (4.40) LysoPA(0:0/18:1(9Z)); ↓ (2.71) PC(22:2(13Z,16Z)/14:0); ↑ (2.25) PE(O-16:1(1Z)/22:6); ↓ (7.29) SM(d18:0/16:0); ↓ (2.71) PC(22:2(13Z,16Z)/14:0); ↓ (17.6) sphinganine; ↓ (2.81) sphingosine; ↓ (2.08) trilinolein	**Lipid metabolism pathway** ↑ (17.4) PE(O-16:1(1Z)/22:6); ↑ (25.5) glucosylceramide (d18:1/18:0); ↑ (7.94) PC(14:0/20:2(11Z,14Z))	**Lipid metabolism pathway** ↑ (19.7) PE(O-16:1(1Z)/22:6); ↓ (2.23) SM(d18:0/16:0); ↓ (10.4) glucosylceramide (d18:1/18:0)

Arrow followed by number in parenthesis indicate direction of change and fold change, respectively.

UDP-GlcNAc, UDP-N-acetylglucosamine; GMP, guanosine monophosphate; UDP, uridine 5’-diphosphate; FAICAR, phosphoribosyl formamidocarboxamide; SM, streptomycin; PC, cytidine monophosphate; PE, O-phosphoethanolamine; PA, adenosine monophosphate; FAD, Flavin adenine dinucleotide.

### Effects of amikacin and meropenem alone or in combination on nucleotide metabolism in *P. aeruginosa*

Antibiotics alone and in combination interfered with nucleotide metabolism at different times and to varying degrees. Within the purine metabolic pathway, several metabolites including deoxyguanosine, guanine, hypoxanthine, inosine, phosphoribosyl formamidocarboxamide (FAICAR), guanosine monophosphate (GMP), and guanosine were identified as being significantly impacted. Amikacin and meropenem group exhibited a substantial reduction in hypoxanthine (log_2_FC=-7.12 to -14.2) and guanine (log_2_FC=-2.85 to -1.57) levels at three different time points. Additionally, amikacin alone led to a decrease in hypoxanthin levels at 4 h (log_2_FC=-1.88), while the impact of meropenem on guanine levels at the three time points was comparatively smaller than that of the combined drug group (log_2_FC=-2.14 to -1.12). Amikacin alone induced a significant increase in deoxyguanosine, FAICAR, and xanthine levels at 1 h (log_2_FC=1.20 to 2.77). The combination of amikacin and meropenem resulted in the decrease of inosine at 15 min and 4 h (log_2_FC=-1.33 and -1.60, respectively). Notably, the combined therapy group exhibited a significant alteration in L-glutamine levels at 15 min (log_2_FC=-1.74), which persisted until 4 h (log_2_FC=-2.07). GMP levels were exclusively affected by amikacin at 1 h (log_2_FC=2.02).

**Figure 4:**
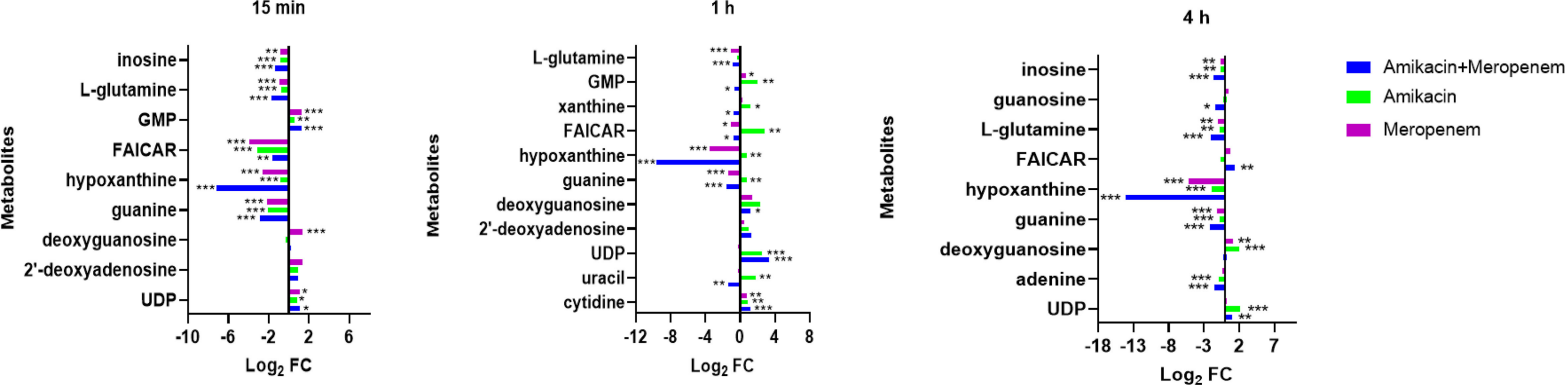
Interference of the nucleotide metabolism pathway of *P. aeruginosa* at 15 min, 1 h, and 4 h, by amikacin and meropenem alone and in combination. Significantly perturbed metabolites were selected based on log_2_FC ≤−1 or ≥ 1, p < 0.05; *p < 0.05; **p < 0.01; ***p < 0.001.

Cytidine, uracil, and uridine 5’-diphosphate (UDP) are associated with the pyrimidine metabolic pathway. Amikacin and meropenem combination group demonstrated a significant reduction in uracil levels exclusively at 1 h (log_2_FC=-1.34). Amikacin induced an increase in UDP levels at 1 h (log_2_FC=2.55), which was sustained until 4 h (log_2_FC=2.18), whereas meropenem did not exhibit a significant impact on the pyrimidine metabolic pathway (**Figure 4**).

### Effects of amikacin and meropenem alone or in combination on amino sugar, peptidoglycan, and central carbon metabolism in *P. aeruginosa*

**Figure 5:**
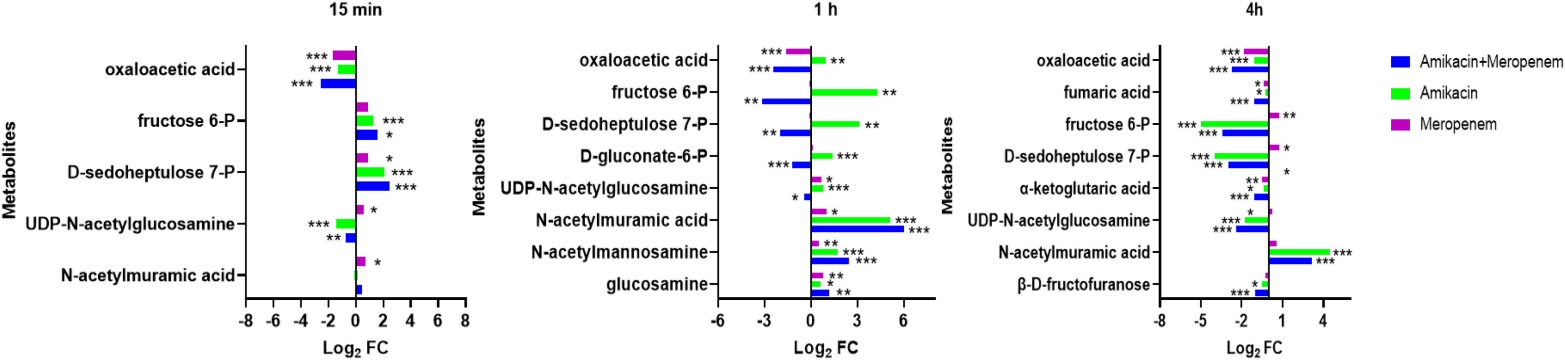
Interference of the peptidoglycan and central carbon metabolic pathways of *P. aeruginosa* at 15 min, 1 h and 4 h by amikacin and meropenem alone and in combination. Significantly perturbed metabolites were selected according to log_2_FC ≤−1 or ≥ 1, p < 0.05; *p < 0.05; **p < 0.01; ***p < 0.001.

N-acetylmannosamine, N-acetylmuramic acid, and UDP-GlcNAc represent metabolites associated with peptidoglycan synthesis (**Figure 5**). At 4 h, amikacin/meropenem combination interfered with the metabolism of these three metabolites. UDP-GlcNAc levels underwent a marked reduction (log_2_FC=-2.39), whereas amikacin demonstrated a relatively milder interference (log_2_FC=-1.79). Both the combined antibiotic group and the amikacin monotherapy induced a notable increase in the abundance of N-acetylmuramic acid (log_2_FC=4.15 and 4.45, respectively).

**Figure 6:**
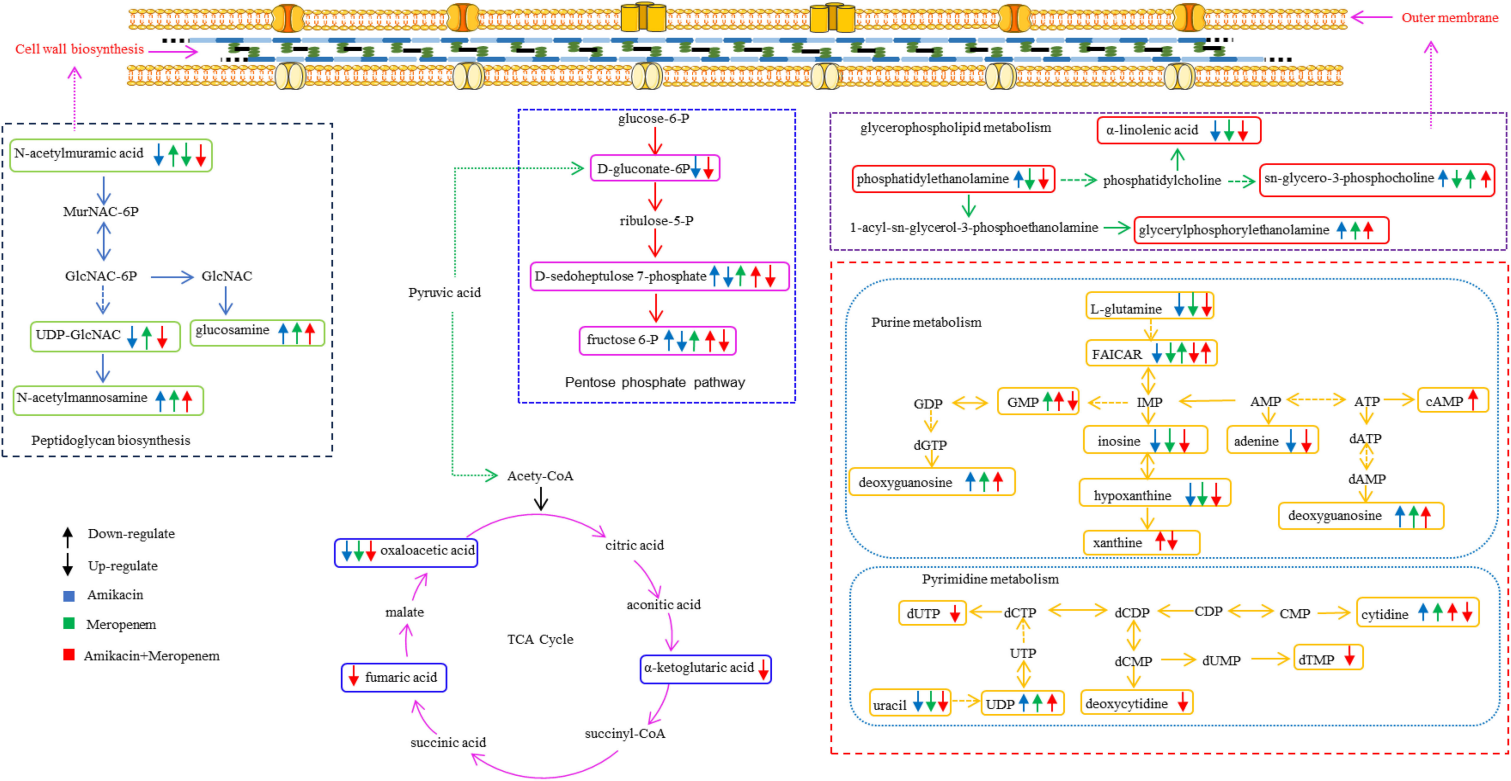
Diagram of metabolic pathways affected by single-agent and combination therapy against *P. aeruginosa*.

The impact on central carbon metabolism affected three compounds that hold critical roles within the tricarboxylic acid cycle (TCA cycle), namely alpha-ketoglutaric acid, fumaric acid, and oxaloacetic acid (**Figure 6**). The combined drug treatment resulted in a decline in alpha-ketoglutaric acid and fumaric acid levels at the 4 h (log_2_FC=-1.02 and -1.11, respectively). The oxaloacetic acid was signficantly reduced across all three timepoints in the amikacin/meropenem group (log_2_FC=-2.7 to -2.52), surpassing the effect exerted by amikacin and meropenem monotherapies (log_2_FC=-1.30 to -1.12 and log_2_FC=-1.84 to -1.69, respectively).

D-gluconate-6-P, D-sedoheptulose 7-P and fructose 6-P are involved in the pentose phosphate pathway. The combination therapy caused a decrease in D-gluconate-6-P levels at the 4 h (log_2_FC=-1.22), while amikacin alone induced an increase (log_2_FC=1.40). D-sedoheptulose 7-P and fructose 6-P levels briefly increased (log_2_FC=2.45 and log_2_FC=1.61, respectively) followed by a reversal at the 1 h and 4 h (log_2_FC=-2.00 to -3.15 and log_2_FC=-2.96 to -3.44, respectively) in the combination therapy, whereas amikacin also resulted in the decrease in these two metabolites after the 4 h (log_2_FC=-3.95 and log_2_FC=-5.00, respectively).

### Effects of amikacin and meropenem alone or in combination on amino acid metabolism in *P. aeruginosa*

**Figure 7:**
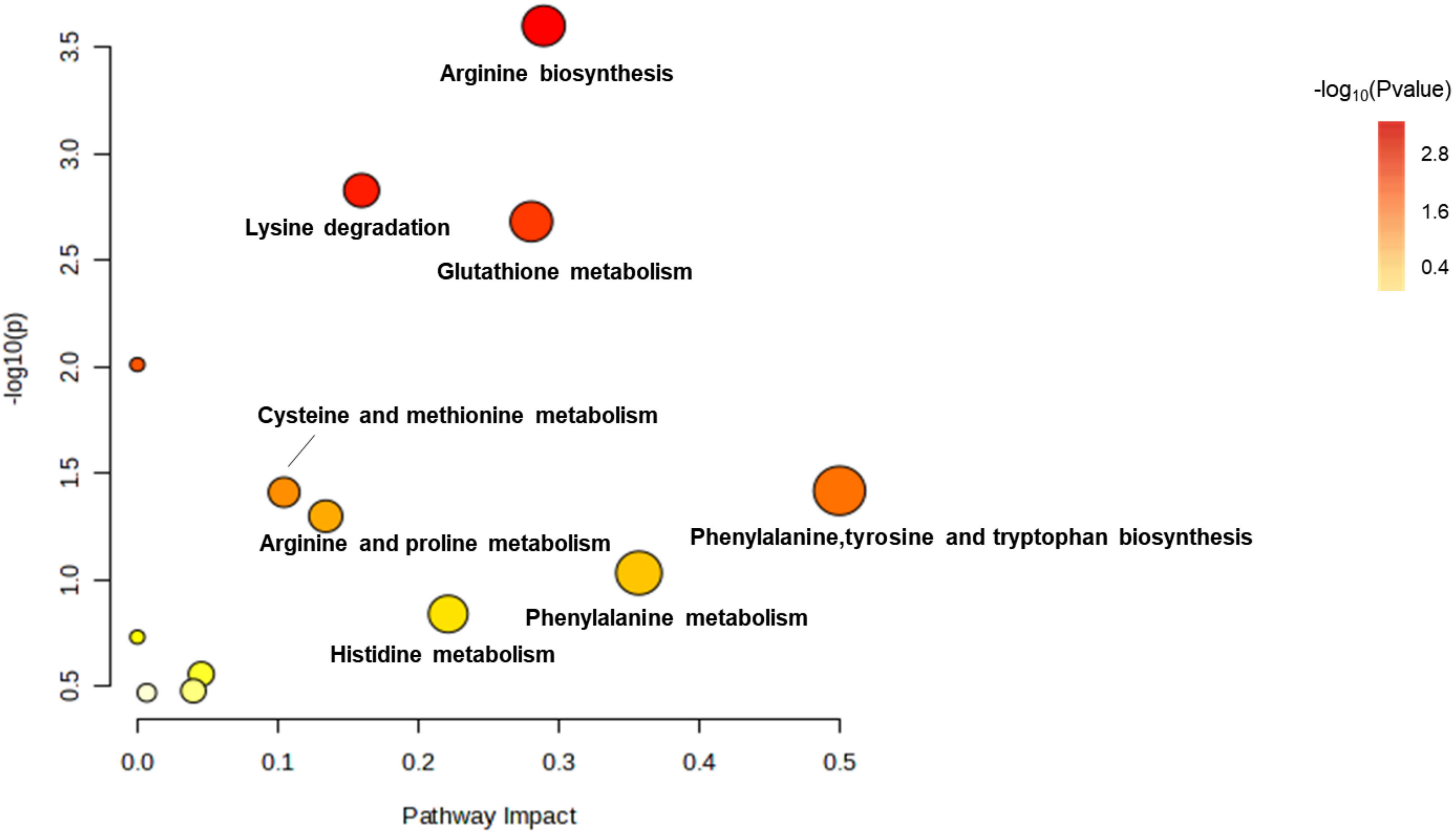
Enrichment bubble plots of amikacin and meropenem alone and in combination showing the disruptiion of amino acid metabolism in *P. aeruginosa*. Significantly perturbed metabolites were selected according to log_2_FC ≤−1 or ≥ 1, p < 0.05.

The levels of arginine, lysine, glutathione, phenylalanine, tyrosine, tryptophan, proline, histidine and phenylalanine were affected by treatments with amikacin and meropenem alone or in combination (**Figure 7**). After 15 min, a significant increase was observed in the levels of L-histidine, malonyl-carnitin, and N-acetyl-L-citrulline across all groups (log_2_FC=1.07 to 1.61, log_2_FC=2.89 to 8.63 and log_2_FC=4.87 to 7.23, respectively). The effect on N-acetyl-L-citrulline persisted for at least 4 h (log_2_FC=4.56 to 7.40). A decrease in N,N-dimethyllysine levels was observed at 15 min with the combination treatment and the change was persistent for over 4 h (log_2_FC=-2.33 to -2.01); the effects of monotherapies diminished by the 4 h. The combination treatment group resulted in a significant decrease in γ-glutamylcysteine at all time points (log_2_FC=-1.01 to -2.01), while meropenem only exhibited a decrease at the 4 h (log_2_FC=-1.05). Amikacin did not exert a significant interference effect on γ-glutamylcysteine.

Amikacin administration led to an elevation in the levels of eight substances: glutathione, L-histidine, N-acetyl-L-citrulline, N-acetylputrescine, N-carbamoylputrescine, N-succinyl-L, L-2,6-diaminopimelate, DL-O-phosphoserine, and 4-aminobenzoic acid (log_2_FC=1.02 to 5.28). The combination treatment group interfered with 15 amino acids, wherein six amino acids had significant reductions (log_2_FC=-3.70 to -1.01), while nine amino acids displayed significant increases (log_2_FC=1.07 to 8.20). The levels of N-acetylputrescine and L-citrulline at 4h were significantly diminished in both the amikacin/meropenem and the amikacin monotherapy groups (log_2_FC=-2.77 to -4.47 and log_2_FC=-2.67 to -2.42, respectively).

### Effects of amikacin and meropenem alone or in combination on lipid metabolism in *P. aeruginosa*

**Figure 8:**
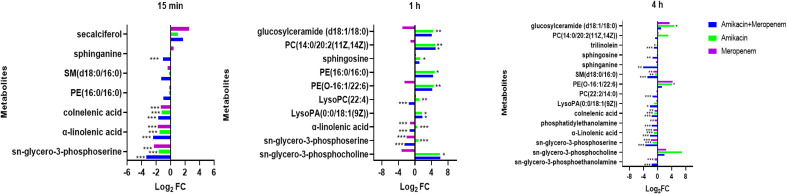
Disruption of amino sugar and lipid metabolism in *P. aeruginosa* by amikacin and meropenem alone and in combination. Significantly perturbed metabolites were selected according to log_2_FC ≤−1 or ≥ 1, p < 0.05; *p < 0.05; **p < 0.01; ***p < 0.001.

As shown in **Figure 8**, the amikacin/meropenem combination significantly reduced the levels of sn-glycero-3-phosphoserine at multiple time points (log_2_FC=-3.46 to -2.50), and the degree of interference was higher than that of meropenem alone (log_2_FC=- 2.31 to -1.96). The interference of each combination on sn-glycero-3-phosphocholine primarily occurred at 1 h and 4 h (log_2_FC=-3.29 to 6.17 and log_2_FC=2.00 to 6.92, respectively), and the combined administration group also reduced the level of phosphatidylethanolamine at 4 h (log_2_FC=-3.13).

Other substances involved in lipid metabolism also experienced significant interference. The antibiotic combination group induced a significant decrease in PE(16:0/16:0) at 15 min (log_2_FC=-1.02), which was reversed at 1 h (log_2_FC=4.39).

LysoPC(22:4) and PE(O-16:1/22:6) were both up-regulated at 1 h after amikacin monotherapy (log_2_FC=1.09 and 4.48, respectively), whereas meropenem treatment resulted in down-regulated PE(O-16:1/22:6) levels (log_2_FC=-2.52). Combination therapy increased LysoPA(0:0/18:1(9Z)) (log_2_FC=1.78), but after 4 h this situation is reversed (log_2_FC=-2.14). Amikacin/meropenem combination affected SM(d18:0/16:0), sphinganine, sphingosine, trilinolein, and PC(22:2(13Z,16Z)/14:0) at 4 h (log_2_FC=-4.14 to -1.06); meropenem monotherapy only interfered with SM(d18:0/16:0) (log_2_FC=-1.16), while the amikacin group did not exhibit any interference with these metabolites.

## Discussion

The World Health Organization (WHO) categorized carbapenem-resistant *P. aeruginosa* as a level 1 pathogen (Horcajada et al., 2019; Kunz Coyne et al., 2022). Amikacin is a primary therapeutic option for combating *P. aeruginosa* infections. However, achieving optimal antibacterial efficacy requires attaining a peak drug concentration of 8 to 10 times MIC of the pathogen when employing monotherapy (Layeux et al., 2010); high-dose regimens can increase the risk of toxicity (Duszynska et al., 2013). The examined isolate in this study harbors the PAO resistance gene, which confers resistance to meropenem (Sumita and Fukasawa, 1996). Antibiotic combination resulted in a reduction of the amikacin MIC from 2-16 mg/L during monotherapy to 0.5-2 mg/L, while the meropenem MIC decreased from 4-16 mg/L to 1-2 mg/L.

The metabolomic studies employed in the current study extend the understanding of the downstream effects of combination therapy responsible for its synergistic activities. Multiple metabolic pathways in MDR *P. aeruginosa* essential for bacterial survival were disrupted more markedly in the meropenem/amikacin combination than single-agent therapy; these metabolites are part of the nucleotide, amino acid, lipid, peptidoglycan and central carbon metabolic pathways, which are summarized in chronological order in **Table 2**.

N-acetylmuramic acid and UDP-GlcNAc that serve as vital constituent in the architecture of bacterial cell walls (Demeester et al., 2018; Dorr et al., 2019) were severely depleted in the combination therapy; the effect was instantaneous compared to that of the single drug group. Phosphatidylethanolamine and sn-glycero-3-phosphoserine, which are essential components of the biological cell membranes (Sohlenkamp and Geiger, 2016; Cho et al., 2021) were downregulated; perturbation in their pathways likely introduced instability of the bacterial cell membrane, allowing for amikacin to freely enter the bacterial cell and exert its effects (Nikaido, 2003; Ruiz et al., 2006; May and Grabowicz, 2018).

The perturbation of several amino acid and nucleotide metabolites likely attributed to amikacin which is known to disrupt bacterial protein synthesis through binding to the 30S ribosomal subunit (Vakulenko and Mobashery, 2003; Dudek et al., 2014). The co-administration of amikacin and meropenem significantly down-regulated the levels of γ-glutamylcysteine, a crucial precursor for glutathione synthesis (Anderson and Meister, 1983; Deneke and Fanburg, 1989). Reduction in glutathione levels below a certain threshold triggers apoptosis signaling, leading to programmed cell death (Franco and Cidlowski, 2009; Circu and Aw, 2012).

This study also showed that combination therapy exerted a more pronounced impact on purine metabolism compared to pyrimidine metabolism. In contrast, the influence of the single drug treatment on nucleotide metabolism was relatively weak, particularly evident at the 4-hour time point, where the perturbed substances were significantly diminished. Nucleotide metabolic pathways play vital roles in bacterial cell metabolism (Lopatkin and Yang, 2021; Goncheva et al., 2022).

Fumaric acid, oxaloacetic acid and α-ketoglutaric acid, which serve as crucial intermediates within the tricarboxylic acid cycle (TCA) (Haddad and Mohiuddin, 2023) critical in cellular energy production, acetyl-CoA provision, and the supply of precursors for various biosynthetic processes (Eniafe and Jiang, 2021), were disrupted by the combination therapy. The production of reactive oxygen species poses a threat to bacterial cell components. To counteract oxidative stress, bacteria employ specific enzymes, such as catalase, and catalase and rely on NADPH. When the combination therapy interferes with the pentose phosphate pathway (PPP) that plays a critical role in maintaining NADPH levels (Ralser et al., 2007; Mishra and Imlay, 2012; Christodoulou et al., 2018), energy equilibrium within the bacteria is destabilized.

In summary, our metabolomic analysis elucidated the impact of amikacin/meropenem combination therapy on the metabolic pathways of *P. aeruginosa*. Our findings demonstrated that this antibiotic combination exhibited a prolonged duration of action and induced more pronounced changes in metabolite abundance compared to the monotherapy, leading to a faster bacteria death.

## Data availability statement

The original contributions presented in the study are included in the article/supplementary material. Further inquiries can be directed to the corresponding author.

## Author contributions

HY: Investigation, Methodology, Resources, Writing – original draft, Writing – review & editing. ZH: Methodology, Writing – review & editing. JY: Methodology, Writing – original draft. JC: Methodology, Writing – review & editing. MY: Writing – review & editing. CQ: Supervision, Writing – review & editing.
